# An Isomorphic Interactive Device for the Interventional Surgical Robot after In Vivo Study

**DOI:** 10.3390/mi13010111

**Published:** 2022-01-11

**Authors:** Cheng Yang, Shuxiang Guo, Xianqiang Bao

**Affiliations:** 1 School of Automation, Beijing Institute of Technology, Beijing 100081, China; yangcheng@bit.edu.cn; 2 Key Laboratory of Convergence Biomedical Engineering System and Healthcare Technology, The Ministry of Industry and Information Technology, Beijing Institute of Technology, Beijing 100081, China; 3 Faculty of Engineering, Kagawa University, 2217-20 Hayashi-cho, Takamatsu 760-8521, Japan

**Keywords:** force feedback, isomorphic interactive device, interventional surgery, master–slave control, robot-assisted surgery, surgical robot controller

## Abstract

Interventional surgical robots are widely used in neurosurgery to improve surgeons’ working environment and surgical safety. Based on the actual operational needs of surgeons’ feedback during preliminary in vivo experiments, this paper proposed an isomorphic interactive master controller for the master–slave interventional surgical robot. The isomorphic design of the controller allows surgeons to utilize their surgical skills during remote interventional surgeries. The controller uses the catheter and guidewire as the operating handle, the same as during actual surgeries. The collaborative operational structure design and the working methods followed the clinical operational skills. The linear force feedback and torque feedback devices were designed to improve the safety of surgeries under remote operating conditions. An eccentric force compensation was conducted to achieve accurate force feedback. Several experiments were carried out, such as calibration experiments, master–slave control performance evaluation experiments, and operation comparison experiments on the novel and previously used controllers. The experimental results show that the proposed controller can perform complex operations in remote surgery applications and has the potential for further animal experiment evaluations.

## 1. Introduction

**Figure 1:**
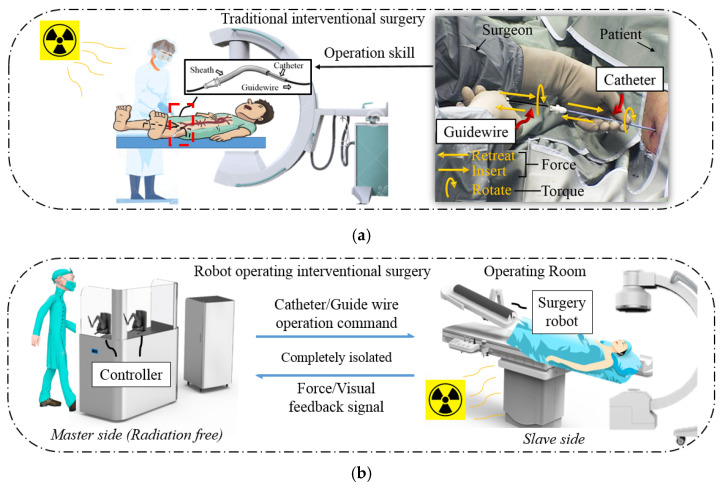
Routine operation procedure of interventional surgery and advantages of robot operation compared to the traditional method. (**a**) Traditional interventional surgery needs to be conducted in the operating room. The surgeon manipulates the catheter with one hand and guidewire with the other. The delivery (insert), withdrawal (retreat), and twisting (rotate) of the catheter and the guidewire are involved when passing through different blood vessels during the operation. (**b**) Robot-assisted surgery can be conducted outside the operating room. The surgeon can be protected from radiation, operate the catheter and guidewire remotely using the robot’s controller.

According to the 2020 world health statistics published by the World Health Organization, cardiovascular diseases caused 17.9 million deaths in 2016, being the most fatal among all non-communicable diseases [1]. The traditional open surgery treatments of cardiovascular diseases are harmful to patients and not conducive to postoperative recovery. Therefore, interventional surgery has recently become the primary treatment of cardiovascular diseases. As shown in Figure 1, during interventional surgeries, surgeons create tiny incisions at the patients’ femoral artery or radial artery and insert a catheter and guidewire for operation. The catheter plays a supporting role during the operation, whereas the guidewire plays a guiding and positioning role. With the help of medical image guidance, the surgeon delivers and rotates the catheter and guidewire such that the catheter and guidewire can branch through the narrow blood vessel and enter the target position (operation detail discussed in Section 2). After reaching the target position, the surgeon withdraws the guidewire and delivers the stent or medication through the catheter. Interventional surgery has the advantage of being minimally invasive and a quick recovery; its disadvantage is that surgeons are exposed to radiation during the surgery. Although surgeons usually wear lead clothes for protection, some operations may take hours; thus, surgeons must remain highly stressed. According to our previous communication with surgeons, the number of operations can reach 18 during the busiest day: “A day’s surgery is equivalent to 1000 chest radiographs.” As a result, interventional surgical robots have been extensively proposed as a solution to radiation problems [2]. The main aim of interventional surgical robots is to allow surgeons to perform surgical operations on patients outside the operating room. Here, the robotic part outside the operating room (i.e., the master side) is required to allow surgeons to remotely issue surgical instructions with their familiar operating techniques, and the robotic part inside the operating room (i.e., the slave side) should operate the surgical equipment based on the instructions. This master–slave structure is currently the main operating structure in studies on surgical robots.

In this study area, the research focus differs between companies and universities or research institutions. Companies initiated the launching of robotic systems for vascular interventional surgery that can be used clinically. Currently, the representative commercial products include Corpath^®^, Sensei^®^X, Magellan™, Amigo™, and Niobe™. Stereotaxis Inc. developed the Niobe™ remote navigation system in 2002. The system is composed of two permanent magnets that generate static magnetic fields from 0.08 to 0.1 T, which are moved around the patient to orientate and steer the catheter remotely [3]. The Corpath^®^ was designed by Corindus Vascular Robotics in 2004. This robot uses friction wheels to control the catheter’s linear and rotational motion. The controllers of the master side are joysticks, and the movements can reach millimeter precisions. However, the system cannot provide force feedback [4]. Sensei^®^X, which was developed by Hansen Medical in 2006, aimed to facilitate a controlled and precise positioning of catheters within the cardiovascular system [5]. The Magellan™ system, which was also developed by Hansen Medical, has verified its operation performance through clinical experiments, and successfully completed stent implantation and aneurysm repair [6]. However, the system cannot provide force feedback, and surgeons can only perform surgical operations under the guidance of two-dimensional images. Catheter Robotics designed the Amigo™ robot system in 2008, providing remote controllers with push buttons on the master side and a multi-freedom steerable catheter controller on the slave side [7].

Universities and research institutions have also studied robotic systems for vascular interventional surgery [8,9,10]. In our previous studies, a novel robotic interventional surgery system was presented. The system can operate a catheter and guidewire in both cooperative and independent operations. The surgery system has performed several animal experiments and clinical trials and has proven its potential in clinical use [11,12,13,14,15]. Other research institutions have also presented their robotic systems. For example, S. Norouzi-Ghazbi et al. at Ryerson University presented a robot-assisted catheterization system that can navigate the catheter tip to a designated target with accuracy exceeding 90% in both velocity and positioning mode [16]. Naveen et al. at the University of Illinois at Urbana-Champaign presented an endovascular robotic system. The robot used force calibration to find a dynamic threshold that elicits haptic vibrations to alert surgeons when applying excessive force on blood vessels via the surgical robot, which is called “adaptive thresholding” [17]. In addition, Howe et al. at the Harvard School of Engineering and Applied Sciences in the United States, Guangzhong et al. at Imperial College London in the United Kingdom, Govindarajan et al. at the State University of New York (SUNY) at Buffalo, and Kouhei et al. at Keio University in Japan conducted related studies on robotic systems for vascular interventional surgery and published a series of papers [18,19,20,21].

Among the various studies in vascular intervention surgery robots, researchers faced problems with studies on master controllers [22]. Master controllers are designed to collect data from surgeons’ operation movements, including linear and rotational motions. For operation safety, master controllers should also provide force feedback. Most of the presented robots either use joysticks or commercial haptic device products as their robot master side. Joysticks such as gamepads can be used to control the catheter and other surgical equipment. However, they lack force feedback. Commercial products such as the haptic interaction device Geomagic^®^ Touch (3D Systems Corp, Rock Hill, SC, USA) can provide data collection and force feedback, enabling them to be used as the master side of interventional surgical robots. However, based on our experience, haptic devices have the following three disadvantages:

First, the operation design of these haptic devices does not fit the actual operating habits of surgeons. Haptic devices are usually multifunctional in a three-dimensional working space. Their operating handle usually has a limited stroke in a single linear direction, which cannot meet the requirement of catheter or guidewire manipulation.

Second, the handles of these haptic devices are not designed for 360° rotations (e.g., Geomagic^®^ Touch X). Surgeons have to achieve continuous rotation through software programming. Meanwhile, the consistency of force feedback and the force detected from the slave side is unknown. Force feedback is performed in open-loop control;

Third, the cost of haptic devices is expensive. The cost of widely used commercial haptic devices ranges from USD1500 (e.g., Geomagic^®^ Touch phantom omni™) to USD20,000 (e.g., Force-dimension Omega.7). For a cooperative operation (detail in Section 2), using two haptic devices for the catheter and the guidewire is essential. Here, the excessive investment in controllers has hindered the development of interventional surgical robot research and commercialization.

**Figure 2:**
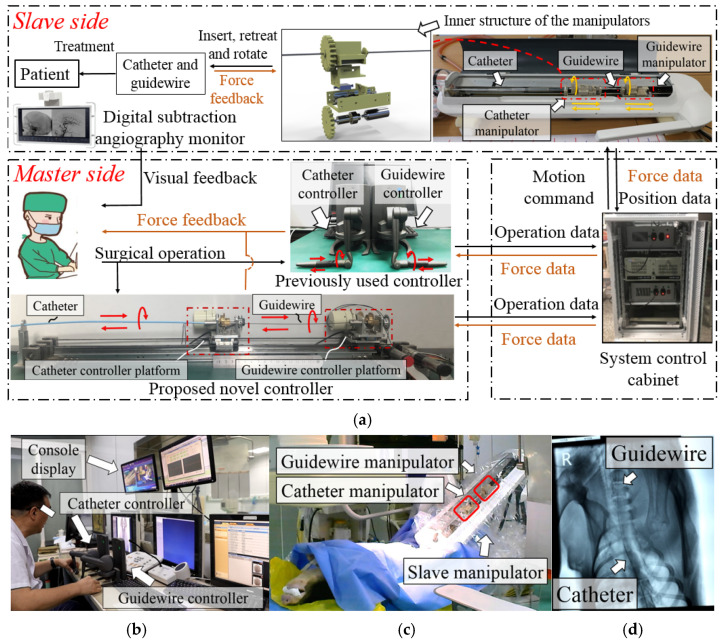
Overview of the developed interventional surgery robot system. (**a**) The concept of master–slave robot-assisted surgery. The surgeon can remotely control the motion of the slave side catheter and guidewire by operating one of the two types of master controllers [15]. (**b**) The operating environment of the master side during previous animal experiments. The surgeon performed intervention operations on an experimental pig outside the operating room. (**c**) The slave side operating environment during previous animal experiments. The slave robot was placed above the operating table, and the catheter and guidewire were inserted into the experimental pig through a puncture. (**d**) Partial angiography results of previous animal experiments [14].

Based on previous studies, our lab developed a novel remote-controlled vascular interventional robot [12,13,14,15]. As shown in Figure 2, this robot can manipulate both the catheter and guidewire, and provide force feedback. The master controller of this robot consists of two identical haptic interaction devices (Geomagic^®^ Touch, 3D Systems Corp, Rock Hill, SC, USA). When the surgeon rotates or linearly moves the handle of the master controller along the red arrow in Figure 2a, the robot system can send the operating data to the slave side through the control cabinet. The corresponding manipulator is controlled to move the same distance. Two Geomagic^®^ haptic interaction devices can control the catheter and the guidewire to move separately or simultaneously for a cooperative operation. A grating ruler is installed at the slave side of the robot, and the movement accuracy of the robot system is controlled by reading the data of the grating ruler. The detailed control strategy is shown in our previously published paper [14]. The force detection sensor is placed inside the catheter and guidewire manipulators. When the catheter or guidewire is bent, deflected, or blocked during an operation, the feedback force will push the bracket (shown in Figure 2a) to move toward the force sensor, which can generate a force signal back to the control system cabinet [15]. The detailed precision evaluation results of the force detection structure are shown in our previously published paper [11]. Several in vivo animal experiments and clinical trials have been performed to validate this robot’s structural design and control strategy, which were conducted by experienced surgeons.

During the feedback discussion with surgeons, the inconsistency of the design of the master and slave sides was mentioned as the emphasis on control defect (discussed in Section 2). Surgeons prefer an isomorphic designed controller to operate the surgical robot, which will assist surgeons in the rapid understanding of control strategies.

This paper presents a novel surgical robot controller with force feedback to enable surgeons’ maximum surgical operation potentials when using surgical robots. The operation method mimics the clinical operating habits of surgeons using catheters and guidewires. The force feedback design improves the safety and stability of surgery under remote operating conditions. The blood vessel model experiment proves that the proposed master side controller can control the slave side robot to complete the interventional operation. The remainder of this paper is organized as follows. In Section 2, the structure of the novel master controller is presented. In Section 3, we describe the primary control strategy of the controller. Section 4 details the control and force feedback accuracy evaluation through high-precision calibration experiments and human vascular model experiments. Discussions are provided in Section 5, and Section 6 summarizes this study and provides the conclusions.

## 2. Robot System Description

The routine operation procedure of the catheter and guidewire in an interventional surgery is shown in Figure 1 [23]. The guidewire is sheathed inside the catheter during the operation, and the ultimate goal of the operation is to deliver the catheter to the target location. In the catheter and guidewire insertion process (both hands can be operated simultaneously or independently), the surgeon may encounter the following two difficulties:

(1) Enter the wrong blood vessel. For the health of the patient and the surgeon, the surgeon cannot always observe the position of the catheter and guidewire inside the patient through x-rays during an operation. Therefore, there may be cases in which the catheter enters the wrong path during the operation. At this time, the surgeon needs to retreat the catheter and the guidewire.
(2) Select the target blood vessel branch. When multiple vascular branches simultaneously appear in the surgical path, the surgeon needs to operate the guidewire to assist the catheter. At this time, the surgeon needs to rotate the catheter and the guidewire, point the curved tip of the guidewire toward the target blood vessel, and deliver it forward. After the guidewire enters the target branch, it supports and guides the catheter into the target branch. The operation at this time requires the coordinated operation of the catheter and the guidewire. This job relies heavily on the clinical surgical skills of the surgeon, and the duration can also be used to judge novices and experienced surgeons.

The surgeon can manipulate the master side to adjust the movements and overcome the difficulties mentioned above when conducting animal experiments. However, during an operation, the surgeon also requires the robot developer to advise on the operation method from time to time. Compared with the direct clinical operation, the robot operation for the same target took longer. Based on the feedback from surgeons, the previously used master controller has the following defects:

(1) The previously used master controller has a limited linear operating range, which cannot meet the catheter and guidewire movement requirement;
(2) The previously used controller cannot operate a 360° rotation in a single movement for the catheter and guidewire;
(3) The control method of the previously used controller differs from the clinical surgery. Therefore, the surgeon cannot apply their catheter and guidewire operation skills in the master side control.

To maximize the operability of the surgical robot and shorten the operation time, we propose a novel robot controller. This section introduces the structure of the master side controller from the following two aspects: the surgical operating detection part and the force feedback structure part.

### 2.1. Master Controller Design Overview

**Figure 3:**
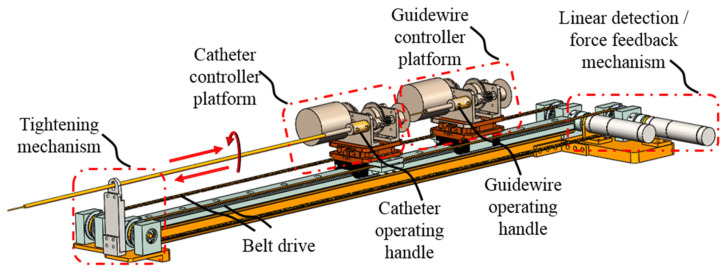
Schematic of the master side controller structure. The operator needs to hold the operating handle of the catheter and the guidewire with each hand during an operation. The linear detection/force feedback mechanism behind the controller consists of two motors. The encoder of each motor is used in providing feedback to the linear detection of each controller platform, and the torque of the motor is used to generate force feedback for the operator.

The novel master controller design overview is shown in Figure 3. It is designed as an isomorphic structure with the slave side robot to assist surgeons in quickly becoming familiar with the robot operation. Two controller platforms (catheter and guidewire) are designed to replace the previous haptic interaction devices (Geomagic^®^ Touch, 3D Systems Corp, Rock Hill, SC, USA). They are placed on a slide rail with a length of 1300 mm, which meets the operation stroke requirements of regular angiography surgery procedures. Surgeons can manipulate each controller platform by operating the controller handle. A catheter and a guidewire are fixed on each controller handle to provide the surgeon with a vivid operating impression during remote control, similar to Figure 1a. Each platform is connected to a linear motion detection motor via a conveyor belt. The linear displacement caused by the movement of the controller handle will drive the motor shaft to rotate through the conveyor belt, such that the encoder of the motor can record the displacement data.

**Figure 4:**
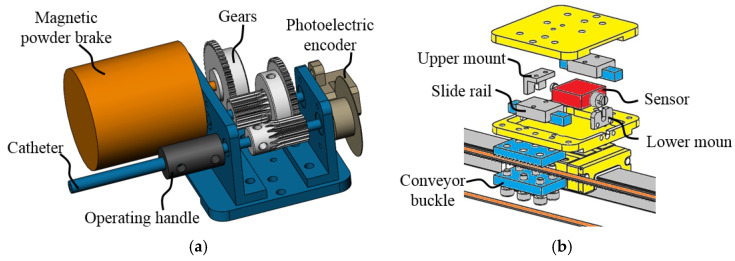
Detailed structure of the controller platform. (**a**) Schematic of the upper controller platform. (**b**) Structure diagram of linear force feedback evaluation mechanism.

The detailed structure of each controller platform is shown in Figure 4a. The two platforms are structurally identical, and the only difference is that the catheter controller handle is fixed with a catheter, and the guidewire controller handle is fixed with a guidewire. The catheter and the guidewire are coaxially sleeved together and follow the operation relationship between the catheter and guidewire in actual interventional surgery. Rotation operation data are captured through a photoelectric encoder.

### 2.2. Force Feedback Structure Design

The main types of force feedback during interventional surgery are divided into linear force feedback (“force” shown in Figure 1a) and torque force feedback (“torque” shown in Figure 1a). When the catheter and guidewire are bent during insertion, or the tip of the catheter and guidewire collide with the vessel wall, the surgeon will feel linear force feedback. When the catheter and guidewire are inserted too long into the patient’s body, the surgeon will feel torque feedback when rotating and adjusting the position of the catheter and guidewire tip.

To realize the interaction of force feedback during a robot operation, the force feedback structure of the novel master controller has been designed in the following three aspects: linear force feedback control mechanism, torque force feedback control mechanism, and linear force feedback evaluation mechanism. The linear force feedback control mechanism is used to realize real-time resistance during the catheter and guidewire advancing operation; the torque force feedback control mechanism is used in providing torque feedback during the catheter and guidewire rotational operation, and the linear force feedback accuracy evaluation mechanism is used to detect the accuracy of linear force feedback, the control system will adjust the linear force feedback in real time based on the detection results.

**Figure 5:**
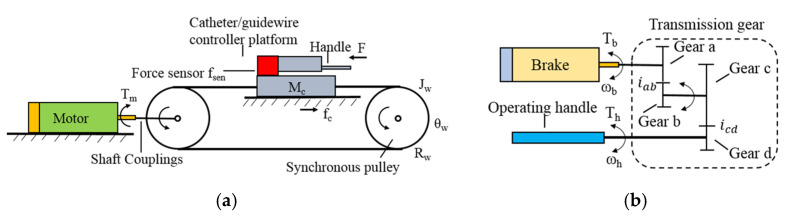
Schematic of force feedback structure. (**a**) Linear force feedback control mechanism. (**b**) Rotation torque feedback control mechanism.

The linear force feedback control mechanism is shown in Figure 3 and Figure 5a. Motors will generate the corresponding torque after the robot system sends a linear force feedback signal by changing the current. Torque is then converted to the resistance force through the timing belt. The torque feedback of the robot is realized by using a magnetic powder brake. As shown in Figure 4a and Figure 5b, when the system receives a torque feedback signal, it generates braking torque by adjusting the excitation current. As the operating handle and the magnetic powder brake are connected through multiple gears, surgeons need to overcome the torque generated by the magnetic powder brake during rotation operation. High-precision torque feedback at different transmission ratios can be achieved by replacing the gear between the operating handle and the magnetic powder brake. The linear force feedback evaluation mechanism is shown in Figure 4b. A force sensor is arranged inside the mechanism, one side of the sensor is connected to the upper platform, and the other side is connected to the lower supporting base. The supporting base is fixed to the conveyor belt through the conveyor buckle. When linear force feedback is generated, the resistance caused by the motor is transmitted to the lower support base through the timing belt. The force sensor can detect the force between the upper platform and lower supporting base since it is the only connection unit.

## 3. System Control Strategy

Upon completion of the structure of the master controller, we use the novel master controller to replace the previous one in robot control. In this section, the control strategy of the novel master controller in the robot system is presented from the following two aspects: the master–slave surgical operation control strategy and the force feedback control strategy.

### 3.1. Surgical Operation Control Strategy

**Figure 6:**
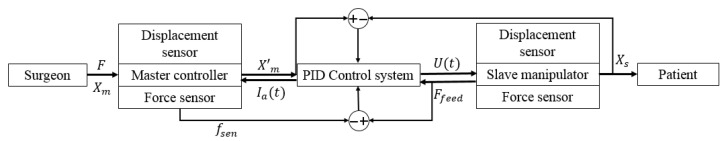
Block diagram of the robot system control strategy.

As shown in Figure 6, the control strategy adopted in the existing robot system is fuzzy PID control to achieve a real-time and high-precision operation. The principle of fuzzy PID control is to determine the fuzzy relationship between the three parameters of PID, the error *e*, and the error rate of change *e_c_*, then adjust the three PID parameters online according to the determined rules of the fuzzy logic. As introduced in Section 1, the preliminary animal experiments and clinical trials proved that this control method could meet the operational requirements of interventional surgery in terms of real-time performance, precision, and safety [14]. For the consistency of the surgical operating system, only the data of motors and photoelectric encoders of the novel master side controller are connected to the existing control system as input in software communication, and the control strategy remains unchanged.

### 3.2. Force Feedback Control Strategy

Regarding force feedback, different control strategies were adopted for linear force feedback and torque force feedback. For linear force feedback, as shown in Figure 5a, the feedback force felt by the surgeon originated from the torque of the motor; therefore, the motor torque model in the locked-rotor state needs to be established.

During operation, the voltage balance equation of the motor armature circuit is given by the following:

(1) $$L_{a}\frac{dI_{a}}{dt}+R_{a}I_{a}=U-E_{a}$$

where *L_a_* is the armature inductance, *R_a_* is the armature resistance, and *I_a_* is the armature current. *U* and *E_a_* denote the armature voltage and induced electromotive force, respectively.

When the motor is functioning in locked rotor mode, the electromagnetic torque of the motor *T_m_* can be given by the following:

(2) $$T_{m}=C_{m}I_{a}$$

where *C_m_* is the mechanical constant, the output torque is only related to the motor current at this time. When the magnetic flux is constant, the motor output torque is proportional to the motor current, thereby completing the feedback force control. The force information detected by the force sensor (shown in Figure 4b and Figure 5a) can be calculated as follows:

(3) $$f_{m}=\frac{T_{m}}{R_{w}}$$

(4) $$F-f_{c}-f_{m}=M_{c}R_{w}{\ddot{\theta }}_{w}$$

(5) $$f_{sen}=F+f_{m}$$

where *f_m_* is the motor force value, *f_sen_* is the force sensor detection value, *F* is the force applied by the surgeon on to the controller platform, *R_w_* and *θ_w_* are the radius and rotation angle of the synchronous wheel, respectively, and *f_c_* represents the resistance force. According to Equations (3)–(5), the force sensor detection value *f_sen_* can be given by the following:

(6) $$f_{sen}=\{\begin{array}{ll} 2f_{m}+f_{c}+M_{c}R_{w}{\ddot{\theta }}_{W} & \left(F\neq 0\right)\\ 0 & \left(F=0\right) \end{array}$$

For operational safety, we only perform force feedback control on the insertion action of the master controller (i.e., *F* > 0), and there is no restriction on the surgeon’s withdrawal operation. As during interventional surgery, the retreat operation of the catheter and guidewire depend on the surgeon’s evaluation. Moreover, the original intention of force feedback is to alert the surgeon to make decisions such as withdrawal. There is generally no danger when retreating the catheter and guidewire in interventional surgery, and the force feedback will retard this action if enabled.

To achieve accurate feedback, compare the sensor data *f_sen_* at the master side with the real-time force feedback signal *F_feed_* sent by the slave side. Based on the difference between the two data, the armature current is adjusted using the PID algorithm, such that the master side motor can trace the slave side force data in real-time. A standard error-based force control law is as follows:

(7) $$f_{a}=F_{d}+K_{f}(F_{d}-f_{e})-K_{v}{\dot{\theta }}_{w}$$

where *f_a_* is the motor actuating force, *F_d_* is the desired force, *f_e_* is the force motor passively applied to the sensor, and *K_f_* and *K_v_* are controller gains. However, this control approach will not work properly for the robotic catheter system because of the limitations of human control. For example, when the feedback force changes, the surgeon’s operation prevents the forces applied to the controller platform from immediately changing. Therefore, a large force tracking error produces an even larger response from the force regulator, resulting in instability or the system entering a limit cycle. To overcome these issues, we proposed a method that uses the current error term to modulate the commanded motor current.

**Figure 7:**
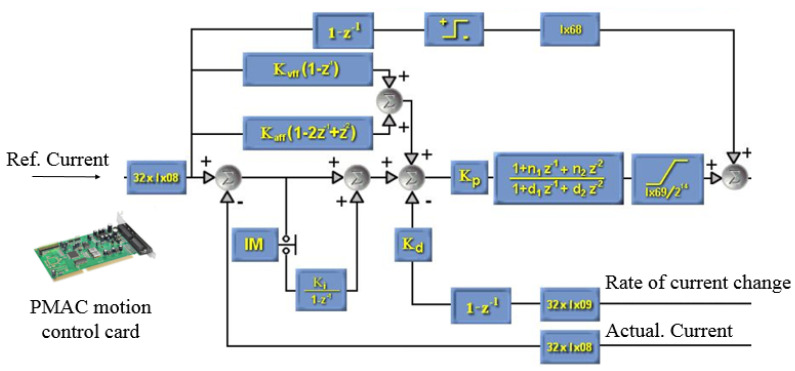
Block diagram of the force control system.

In this force control approach, the master motor is commanded to follow the desired current, *I_a_*, the sum of the current of the motor, *I_e_*, and the current offset required to track the desired force, *I_f_*.

(8) $$I_{a}=I_{e}+I_{f}$$

(9) $$I_{f}=\frac{F_{feed}}{K_{e}}+K_{vff}(F_{feed}-f_{m})+K_{aff}\int \left(F_{feed}-f_{m}\right)dt\;\;\;(F>0)$$

where *K_vff_* and *K_aff_* are controller gains and *K_e_* is the approximate stiffness of the environment. A programmable multi-axis controller (Delta Tau, Fishers, IN, USA) is used in the robot system to actuate the motor’s force feedback control. Through multiple system identification experiments, the corresponding parameters are adjusted in the programmable multi-axis controller program to determine the value of *K_vff_* and *K_aff_* in advance. This control law is similar to the method presented by Eppinger et al. [24]. The block diagram of this controller is shown in Figure 7, where *K_p_* (Ix08; Ix09) is the proportion gain, providing an output proportional to the follow error. The larger the proportional is, the greater the rigidity will be. *K_d_* is the differential gain, and its role is to subtract a number from the current being measured, providing the system enough damper. *K_i_* is the integral gain, decreasing the errors due to the time integral. *K_vff_* is the force feedforward gain; it can add a number to the output of the controller as well as decrease the errors due to the differential gain. *K_aff_* is the force acceleration feedforward gain; it can add a number that is proportional to the desired force acceleration to the output, reducing the follow errors. Ix68 can help to overcome mechanical friction and have no defect on stability. Ix29 is a correction value between the drive output and control card output, which is used to limit the maximum output current value [25].

For torque force feedback, as shown in Figure 5b, the force feedback felt by the surgeon originates from the torque of the magnetic powder brake. As the magnetic powder brake is coaxial with gear A, gear B, and gear C, and gear D is coaxial with the operating handle, the output of the magnetic powder brake can be expressed as follows:

(10) $$T_{h}=i_{ab}i_{cd}T_{b}$$

(11) $$\omega_{h}=\frac{\omega_{b}}{i_{ab}i_{cd}}$$

where *T_b_* is the output torque of the magnetic powder brake, *T_h_* is the output torque on the operation handle, which is the torque felt by the surgeon’s hand. $\omega_{b}$ is the angular velocity of the magnetic powder brake and $\omega_{h}$ is the angular velocity of the surgeon’s rotating operation.

The torque feedback device proposed in this study has not been presently used in actual surgical control since no catheter or guidewire torque sensor was installed on the slave side of the surgical robot. It will be used after the slave side torque feedback is realized in the future.

## 4. Evaluation Experiments and Results

Upon completion of the design of the master controller, it is necessary to improve the accuracy of the controller force feedback and evaluate the surgical performance of the robot system. In this section, the accuracy of the force feedback under the eccentric operating handle was compensated. The performance of the force feedback was verified through static and dynamic experiments. A human blood vessel model experiment was conducted to simulate actual angiographic intubation conditions and to verify the operation fluency and safety of the novel master–slave operating robot.

### 4.1. Eccentric Force Compensation

As shown in Figure 3, because the operating handle of the device is not installed on the axis of the operating platform, it is necessary to consider the force output of the operator and force feedback accuracy in an eccentric condition. To achieve accurate force feedback, a dynamic force test is used to establish a fitting relationship between the eccentric operating force and the axial force sensor data, and its accuracy is verified by static measurements.

**Figure 8:**
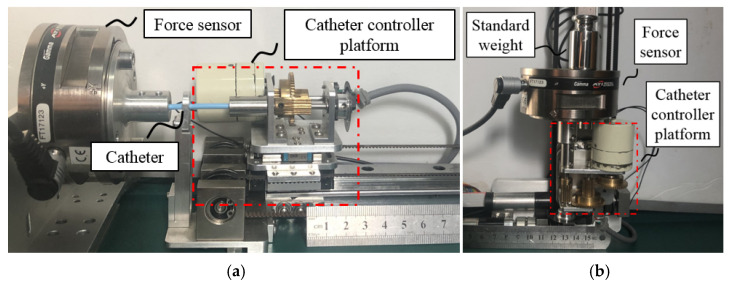
Force feedback accuracy regulation and evaluation. (**a**) Eccentric force compensation and passive force feedback evaluation setup. (**b**) Eccentric force compensation accuracy evaluation setup.

As shown in Figure 8a, the operating handle of the master controller is mounted with a force sensor (Gamma, ATI Industrial Automation, Inc., North Carolina, USA). The movement of the master side platform is operated toward the ATI force sensor to examine the force feedback accuracy in the case of non-coaxial transmission.

**Figure 9:**
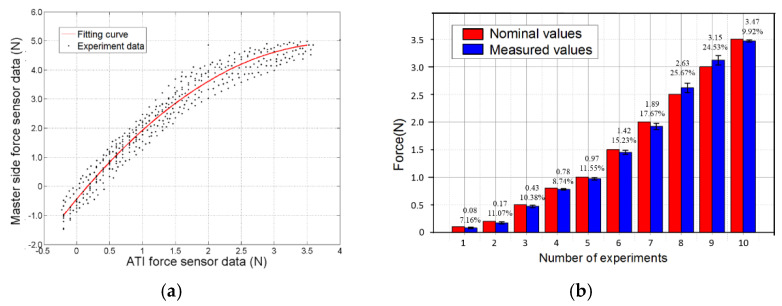
Force calibration experimental results. (**a**) Relationship between MF and AF. (**b**) Evaluation result of fitting accuracy in non-coaxial force transmission. The data displayed on the measured value are the average value and standard deviation based on multiple measurement results.

The relationship between the master side force sensor (M.F.) and the ATI force sensor (A.F.) is shown in Figure 9a. In this figure, the black dots show the relationship between the two force data under the dynamic experiment. The red fitting curve is constructed using MATLAB, and the equation of the curve is as follows:

(12) $$f_{MF}=-0.3409{f_{AF}}^{2}+2.705f_{AF}-0.44$$

As shown in Figure 8b, to verify the accuracy of these fitting results under the dynamic experiment, nominal weights are used to simultaneously perform a static force measurement on the ATI force sensor and the force sensor of the master side. In the experiment, the measured force change of the master side force sensor (*f_MF_*) is recorded. From (12), the measured value of the applied weight can be calculated. By comparing the measured value with the nominal value of the weight, the fitting accuracy of non-coaxial force transmission can be evaluated. As shown in Figure 9b, ten sets of static experiments with nominal values of 0.1, 0.2, 0.5, 0.8, 1.0, 1.5, 2.0, 2.5, 3.0, and 3.5 N were conducted using different weights. The maximum relative error (compared to nominal values) in the experimental results is 10.39%, and the minimum relative error is 5.47%.

Therefore, in actual control, for the operator to feel the force of the slave side *F_feed_*, it is necessary to compensate for the eccentric force and convert the force feedback value *F_feed_* to *f_feed_*. The relationship between the two is as follows:

(13) $$f_{feed}=-0.3409{F_{feed}}^{2}+2.705F_{feed}-0.44$$

The eccentric force can be compensated to make the force feedback in the case of non-coaxial transmission more accurate, allowing the operator to judge the state of the catheter and guidewire from the slave side more precisely, thus improving the safety of the operation.

### 4.2. Controller Passive Force Feedback Accuracy Evaluation

The experimental setup of the passive force feedback accuracy evaluation is similar to the force compensation experiment shown in Figure 8a. During the accuracy evaluation experiment, the operator moves the controller platform forward by holding and pushing the ATI force sensor instead of the operating handle. By comparing the readings of the ATI force sensor, ten sets of static force measurement experiments were performed on the force feedback conditions of 0.5, 1.0, 1.5, 2.0, 2.5, 3.0, and 3.5 N. The experimental results are shown in Table 1.

**Figure 10:**
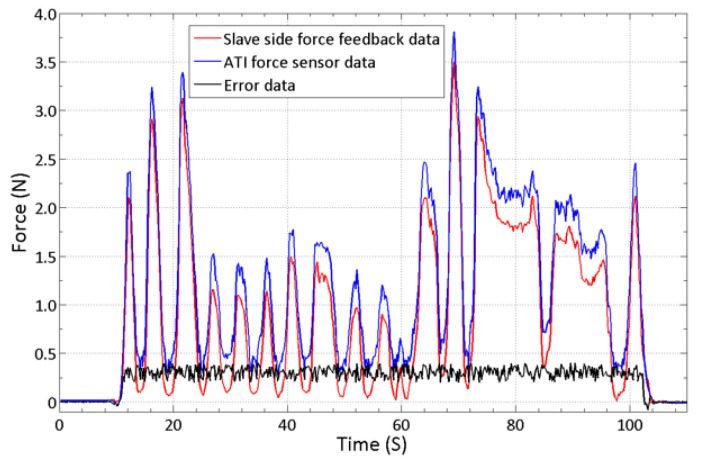
Results of the time-varying force measurement.

**Figure 11:**
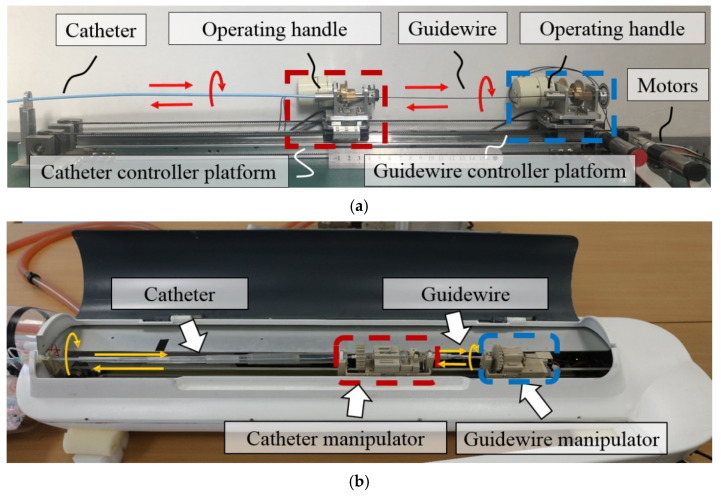
Operational evaluation experiment setup. (**a**) Prototype of the proposed master controller. The main structure of the controller is made of aluminum alloy, and the catheter and guidewire are the same equipment use in actual operation. (**b**) The slave robot being operated at the evaluation experiment. The catheter manipulator was operated by catheter controller platform, the guidewire manipulator was operated by guidewire controller platform.

To verify the accuracy of the force feedback under continuous changes, we performed time-varying force measurements by obtaining the slave side force feedback data. The experimental results are shown in Figure 10. As shown in Figure 10, the proposed controller can generate force feedback and follow the time-varying data when the force feedback data from the slave side (manipulators shown in Figure 11b) changes periodically. However, owing to the thrust generated by the human hand and the operating hysteresis during measurement, the force collected by the ATI sensor during pushing exceeds the actual feedback force from the slave side. In the time-varying force experiment, the maximum relative error and the average relative error between the ATI sensor and slave side force signals are 17.25 and 9.23%, respectively.

As shown in Table 1, in the static force experiment, owing to the limitation of the eccentric structure and motor current control, the passive force feedback data received by the operator have an average relative error of 7.2–9.8%. According to the results of previous studies, the feedback force within the range of 0.5–200 N and the feedback error within the range of 7–10% can meet the requirements of the feedback device [26,27,28,29,30]. In the preliminary animal experiment, the force feedback value exceeding 3 N has been defined as a dangerous operation that must be locked immediately. Based on static force measurement results, we may agree that the passive force feedback is sensitive enough to meet the haptic operation demand.

### 4.3. Robot System Performance Evaluation and Result on a Vascular Model

**Figure 12:**
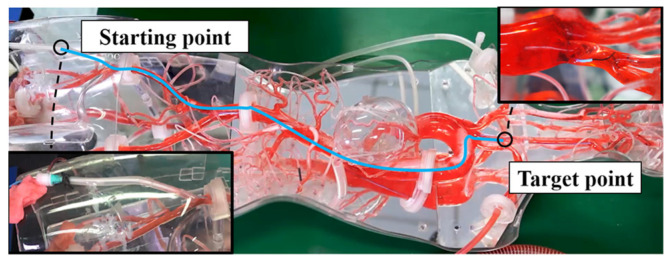
Experiment setup of the vascular model. Including the starting position (the femoral artery) and the target position (the left subclavian aorta).

**Figure 13:**
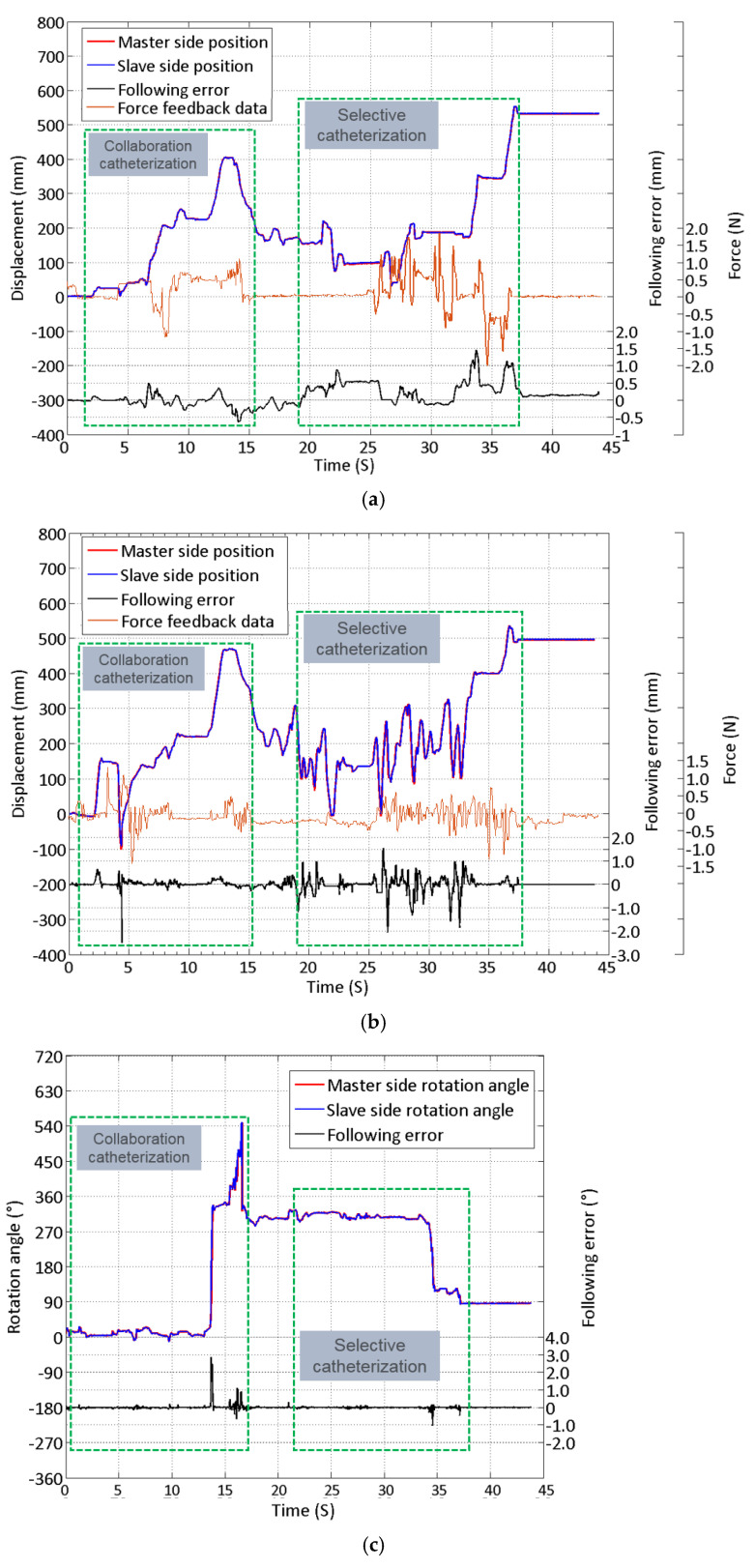

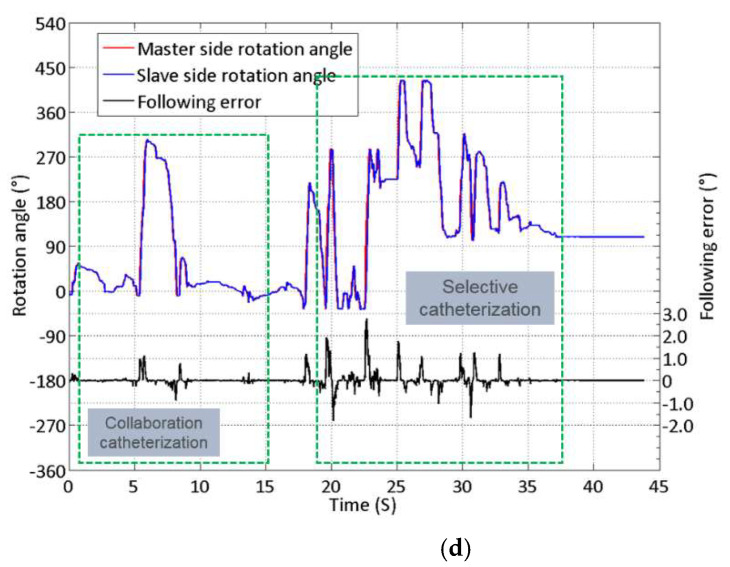
Linear motion, rotational motion result, and the following error of the catheter and guidewire. (**a**) Linear motion result and the following error of the catheter. (**b**) Linear motion result and the following error of the guidewire. (**c**) Rotational motion result and the following error of the catheter. (**d**) Rotational motion result and the following error of the guidewire.

The remote intervention experiment was conducted using a vascular model to evaluate the proposed master controller operation accuracy in the catheter and guidewire linear and rotational motions. The human blood vessel model used in the experiment is a commercial product based on real human blood vessels. It has a pressure, flow, and temperature simulation circulatory system, which can reproduce the human blood circulation during the experiment. The experiment uses the proposed controller (as shown in Figure 11) as the master side and the slave manipulator of the previously developed robot system (as shown in Figure 2a) as the slave side. The overall operation route of the experiment is shown in Figure 12. The experiment used a 5F angiographic catheter with an outer diameter of 1.67 mm and a matching angled guidewire. The operator inserts the catheter and guidewire into the blood vessel of the model through the femoral artery incision, and the target location of the experiment is the left subclavian artery. The difficult part of the experimental operation is to use the controller manipulating the catheter and guidewire to select the left subclavian artery at the position of the aortic arch. As the innominate artery, the left common carotid artery, and the left subclavian artery above the aortic arch are close to each other and are bending in the opposite direction from the aortic arch, it is necessary to repeatedly operate the guidewire back and forth and rotate to enter the target position during the experiment. In the ten effective experiments completed, both the catheter and the guidewire successfully reached the target position. Figure 13 illustrates one of the experimental results of linear and rotational motions of both the catheter and guidewire during evaluation.

As shown in Figure 12, owing to the broader blood vessels in the path from the femoral artery to the aortic arch, this part of the interventional operation was relatively fast. It can be seen from Figure 13a,b that at approximately 15 s, the operator used the robot system to operate the catheter and the guidewire passed through the aortic arch and entered the ascending aorta. Since the complexity of the catheterization path is relatively low, the functions of the cooperative operation are mainly verified in this section of operation, including the use of the novel master controller operating catheter and guidewire for advance, retreat, and rotation. It can be seen from the experimental results in Figure 13 that the proposed master controller can complete these operations both individually and collaboratively. Starting from 13 s, the operator retreated the catheter and guidewire to the descending aorta and started the selection to get into the left subclavian aorta. During an interventional surgery, to make the catheter enter the curved and narrow blood vessel, it is necessary to first manipulate the guidewire into the target position and then use the guidewire to guide the catheter. This kind of operation of selecting specific arteries or even more narrow blood vessels is called selective catheterization and super-selective catheterization. By completing this kind of simulation operation, it can be proven that the novel master controller can be applied to high-precision interventional surgery and has the potential to complete cerebrovascular interventional operations. From 18 to 35 s, the operator mainly performed a repeated insertion and rotation to select the left subclavian aorta from the upper three branches using the guidewire. As the blood vessels here are narrow and curved, it is necessary to use a guidewire to enter the target point first, and then guide the catheter in. This process is reflected in the selective catheterization part of Figure 13. At approximately 36 s, the guidewire entered the target blood vessel, and the operator inserted the catheter into the left subclavian artery, thus achieving the purpose of the evaluation.

As the stiffness of the catheter is comparatively more significant than the guidewire, the average force feedback generated during the operation is higher than that of the guidewire. The catheter linear tracking performance error is between 1.5 and −0.5 mm, and the average error is 0.49 mm; the following error of linear tracking performance of the guidewire ranges from 1.5 mm to −2.6 mm, and the average error is 1.36 mm. The error of the rotation motion of the catheter is between 3° and −0.9°, and the average error is 0.18°; the error of the rotation motion of the guidewire is between 2.8° and −1.8°, and the average error is 0.47°. According to the surgeons’ feedback in the surgery robot’s preliminary animal trial, this error is within the acceptable range during the surgery.

The novel controller and the previously used controller (Geomagic^®^ touch) compared the advantages and disadvantages by performing the same operation experiment on the human model. The operation’s starting point and target point on the human model were the same as in Figure 12. The operators of the experiment were divided into the following two groups: five novices and five experts. The experiment was divided into the following three categories: novices operate two types of controllers; experts operate two types of controllers; experts operate the novel controller without force feedback. The novices here are not ignorant of interventional surgery but have never used any tools to operate. The experts understand how interventional surgery is performed clinically and have used catheters and guidewires to simulate operations on human models. In each experiment, the two controllers were operated ten times, respectively. The operation time, operation stroke, and average force feedback value in the experiment were analyzed in the experiment results.

**Figure 14:**
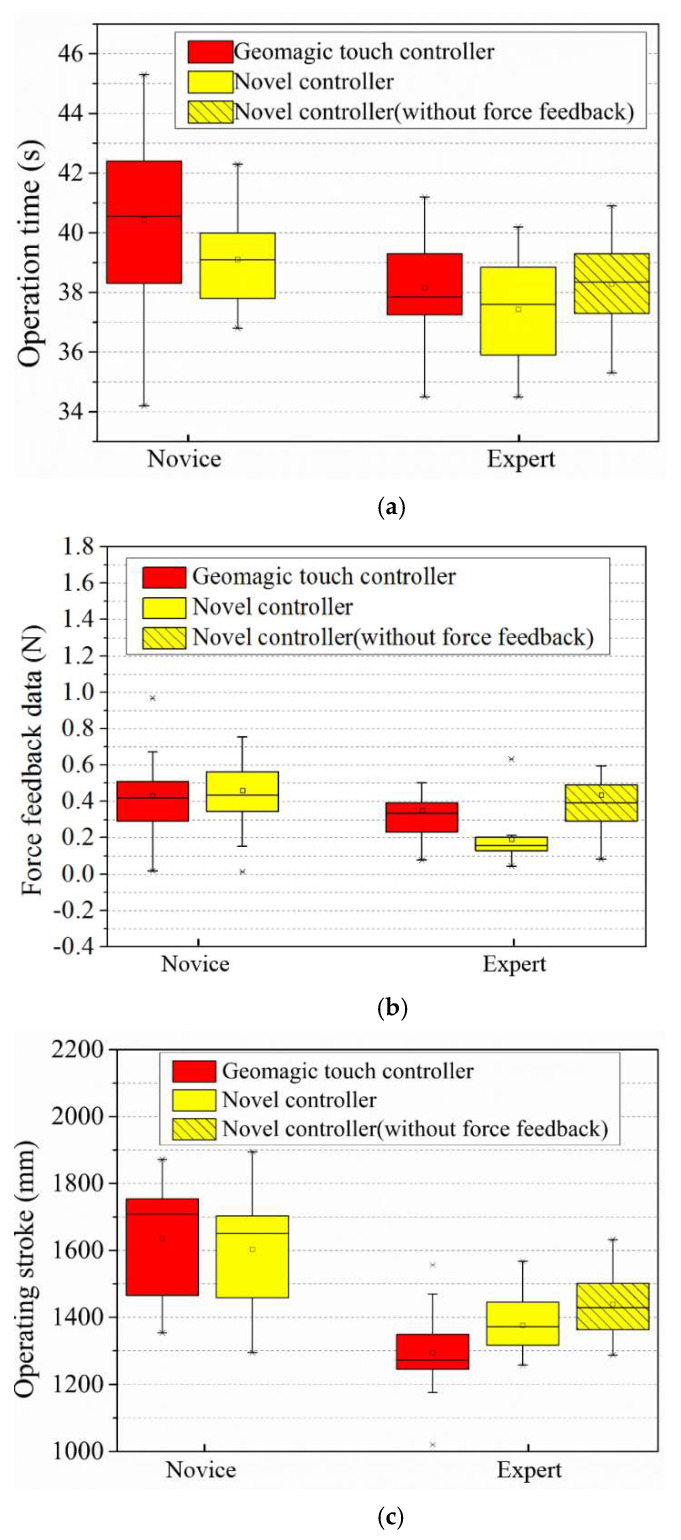
Experimental results of the comparison between the operation of the previously used controller and the novel controller. (**a**) Comparison results of the operation time. (**b**) Comparison results of the force feedback data. (**c**) Comparison results of the operation stroke.

As shown in Figure 14, compared with novices, the experts achieved faster operations, shorter operating strokes, and higher operating safety (lower average force feedback data) when performing surgical operations. As for a novice, it can be found that the experiment operating stroke and operating feedback force have similar results. In terms of operating time, it took less time for the novices to operate the novel controller than the previously used controller to complete the experiment. Therefore, it can be proved that the operation complexity of the novel controller is lower, and the operator can become handy more quickly. Regarding expert operating time, the average time for experts to complete the experiment using the novel controller was 0.78 s faster than using the previously used controller. It is also shown in Figure 14a that the box plot lower quartile statistic of the expert novel controller is lower than the previously used controller. This result means that the novel controller has a higher operational potential: the operator is sure of further shortening the operation time after becoming familiar with it. The experts operating the previously used controller had a shorter operating stroke compared to the novel controller. However, combined with the operating time, experts who used the novel controller completed the experiment faster. This is because the novel controller is more suitable to perform interventional surgery operations; thus, operators are more willing to adjust the position of the catheter and guidewire through surgical actions (insert, retreat, and rotate) when encountering problems during the experiment. In the case of force feedback and no force feedback, the operation time of experts and operation stroke of the human model experiment using the novel controller was similar; however, the average force feedback value was quite different. Regarding surgical safety, force feedback is necessary for the control of interventional surgical robots.

## 5. Discussion

As shown in Table 1 and Figure 13 and Figure 14, the performance of the proposed interventional robot controller force feedback and the accuracy of the control strategy are evaluated. We believe that operation errors occur because of the following:

(1) On the master side, when the controller performs a reciprocating operation, the following errors will occur due to the master–slave transmission delay. This is more significant when the guidewire is frequently operated, such as the data in Figure 13b,d. It can be seen that the rapid operation of 20–35 s caused a large amount of follow-up error in both linear and rotational motion.
(2) On the slave side, because the catheter and guidewire at the slave side will bend or deflect during operation, they may not move according to the expected operation instructions, which will cause difficulties for the surgeon to operate.

Based on previous studies, this study focused on two aspects of the novel master side controller. First, we designed the passive force feedback method according to the proposed controller structure. As the controller handle is eccentrically placed, we conducted compensation experiments to ensure that the operator sensed a more accurate feedback force during operation. Second, we evaluated the master–slave control performance of the controller through a human model. The proposed experimental setup does not account for external or internal disturbances. However, it should be noted that there will be some interference in an actual operation that will influence the performance during operation. For example, the trembling of the arms and fingers of the operator during operation may cause the effect of force feedback to deteriorate. Owing to the difference between the pump speed and composition of the liquid in the human model and the actual blood, the force feedback experience during the test will differ from that of the animal and clinical trials. In terms of function, it is verified that the master side and slave side are isomorphically designed; the operation of the catheter and guidewire can be actuated when using the novel controller.

The system still has some shortcomings after this study: First, because the motor is used for passive force feedback, it works in the locked-rotor state and generates heat. Tests have shown that the performance of the motor will be influenced after a continuous locked-rotor operation for approximately 5 min and affect the accuracy of the encoder’s readings. Second, because the robot is not equipped with a torque sensor at the slave side, it is impossible to verify the performance of the magnetic powder torque feedback structure of the proposed master side controller. Third, the damping environment generated by the designed passive force feedback takes effect during both forward and backward operations, preventing the operator from making timely adjustments after being aware of the force feedback. The operator still receives feedback resistance when performing operations that are opposite to the previous direction of motion. Fourth, compared to the mature force interaction products (Geomagic^®^ Touch, 3D Systems Corp, Rock Hill, SC, USA), the operator is also dragging the timing belt and the connected motor when operating the proposed controller, which will cause a sense of hysteresis even when force feedback is not taking effect.

## 6. Conclusions

In this paper, a novel interventional surgical robot master controller is proposed. The development of this controller fulfills the operation requirements proposed by the surgeon in the preliminary in vivo experiments. It allows the operator to complete the angiographic operation using the clinical operation method remotely. Comparative experiments show that using the novel controller can complete the angiography operation faster than the previously used controller. The force feedback device can reduce the risk of the catheter and guidewire colliding with the blood vessel and bending during a remote operation, reduce the average force feedback data, and improve operation safety. The performance of the controller was evaluated through a vascular model experiment. The results demonstrated that the proposed novel controller could perform remote surgeries in clinical applications. This study provides the following two foundations for our future research in surgical robots:

(1) Proposed an isomorphic master controller to control the interventional surgical robot, mimicking the surgeon’s actual operation of the catheter and guidewire; thus, surgeons can apply their surgical skills during an operation.
(2) A primary force feedback control method was designed to improve the safety of the operator’s remote operation.

In future research, we will focus on solving the above-mentioned problems of the master controller: First, we will attempt to install film pressure sensors on the rotating structure of the slave robot to detect torsion information. Through the magnetic powder brake on the master side, the torque information from the slave side can be used for torque feedback. Second, to optimize the structure of the novel controller, use a grating ruler to collect motion data instead of using the timing belt to avoid hysteresis. Third, a one-way damping control algorithm will be designed according to the surgeon’s operating requirements. The force feedback will be released when the doctor is detected to perform a reverse movement, ensuring the operation’s safety. Finally, the experiment performed in the vascular model did not fully evaluate the operational performance of the proposed master controller. With the help of the partner hospital, we will seek opportunities to use the novel controller in animal experiments and evaluate and improve the controller’s performance through feedback from the surgeon after the operation.

## Figures and Tables

**Table 1:** Static force measurement results using force compensation.

Force (N)	Maximum Error (N)	Average Error (N)	Maximum Relative Error	Average Relative Error
0.5	0.071	0.049	14.2%	9.8%
1.0	0.112	0.082	11.2%	8.2%
1.5	0.151	0.118	10.1%	7.9%
2.0	0.207	0.174	10.4%	8.7%
2.5	0.221	0.180	8.8%	7.2%
3.0	0.258	0.231	8.6%	7.7%
3.5	0.419	0.312	12.0%	8.9%

## Data Availability

The data presented in this study are available on request from the corresponding author. The data are not publicly available due to potential patent application.
